# Supplementary material for: Functional Niche Partitioning Occurs over Body Size but Not Nutrient Reserves nor Melanism in a Polar Carabid Beetle along an Altitudinal Gradient
Source: Insects. 2023 Jan 25;14(2):123. doi: 10.3390/insects14020123 (PMC9967798; doi:10.3390/insects14020123)
Supplement: Supplementary file 1 [file insects-14-00123-s001.zip › insects-2043450-supplementary.pdf]

Principal component analysis (PCA) of the morphometric traits measuring the body size of *A. pacificum*

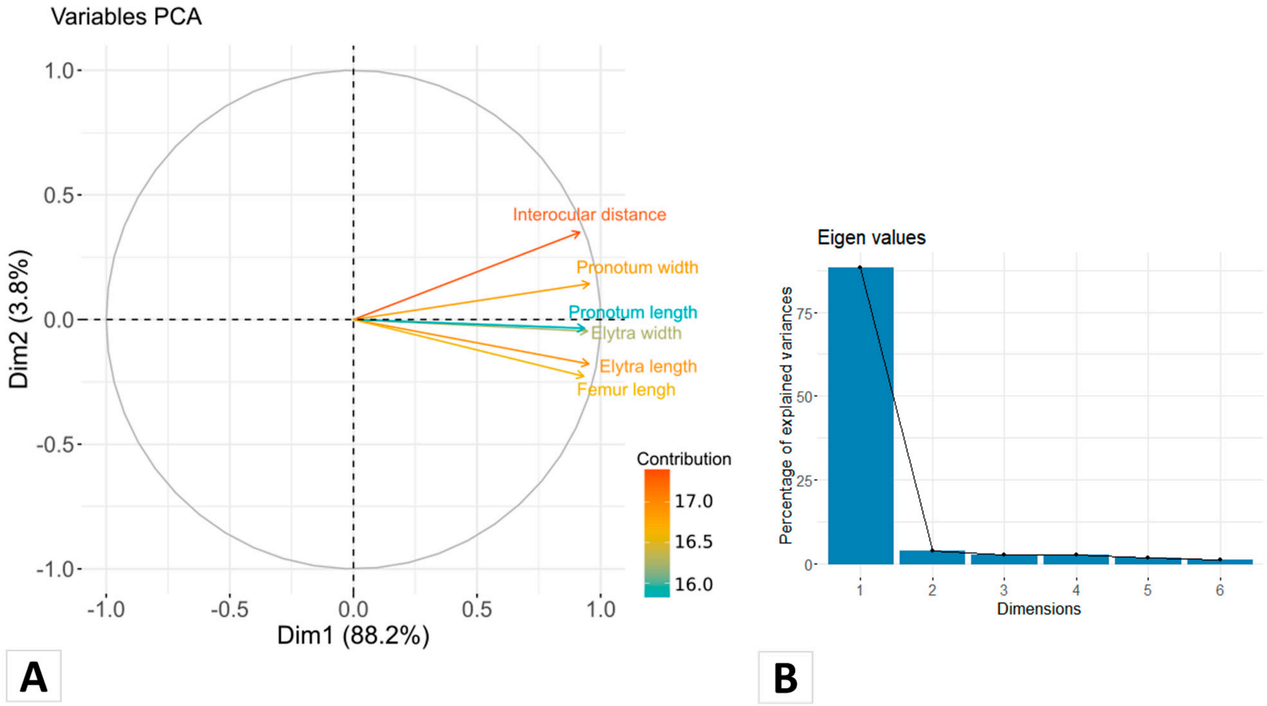

Figure S1. Principal component analysis (PCA) matching the six measured morphometric traits expressing the body size of *A. pacificum*: interocular distance, length and width of pronotum and elytra, length of the femur of the third left leg: **A**-Correlation circle where variables were coloured according to their contribution to the PCA plane; **B**-Eigenvalue histogram of the PCA analysis.

FAMD analysis

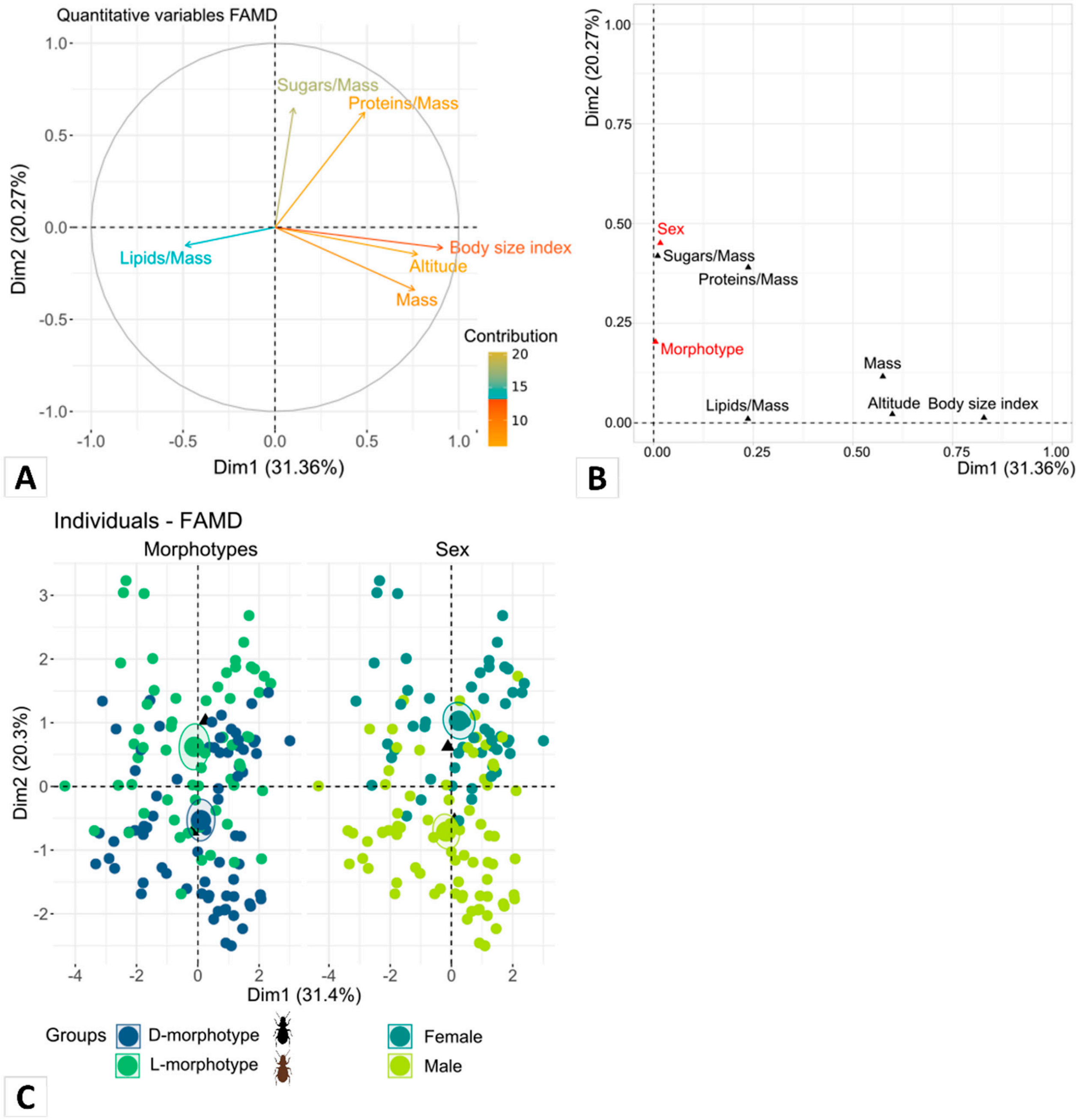

Figure S2. Graphical results of the factor analysis of mixed data (FAMD): **A**- Correlation circle showing quantitative variables; **B**- Biplot showing correlations between both quantitative (black color) and qualitative (red color) variables and the two principal components; **C**- Individual factor maps. Individuals (sorted patterned ground areas) are colored according to morphotype and sex.

The first two principal components of the FAMD accounted for 51.63% of the total variance (Figure S2). The first component (31.36% of the explained variance) was strongly correlated with altitude, Body mass, Body Size index and Lipid amounts, while the second component (20.27% of the explained variance) was strongly correlated with morphotype, sex, sugar amounts, and, to a lesser extent, with protein amounts (Figures S2A-B, Table S1).

Table S1. Contributions of variables (expressed in percentage) to the principal components of the factor analysis of mixed data (FAMD).

Variables	PC1	PC2	PC3	PC4	PC5
Altitude	23.879	1.328	0.066	8.803	0.191
Mass	22.926	7.162	7.007	1.672	4.064
Body size index	33.041	0.768	0.726	0.264	0.062
Proteins/Mass	9.464	24.035	2.334	0.000	4.638
Sugars/Mass	0.407	25.806	7.122	41.480	4.890
Lipids/Mass	9.433	0.598	34.390	1.319	47.011
Morphotype	0.184	12.512	47.233	0.870	35.716
Sex	0.667	27.789	1.121	45.591	3.428

The results showed that individuals from higher altitudes differed from those of lower altitudes in terms of body size, body mass and lipid amounts. Morphological metrics and altitude were positively correlated, and both were negatively correlated with lipid amounts (Figure S2A).

Both sexes and morphotypes seemed to only differ in their biochemical features, and there was no distribution pattern specific along the studied altitudinal gradient (Figure S2B). Analyzing the distribution of individuals on the FAMD plane according to their sex (Figure S2C) showed that most of the females may store more sugars and proteins than males, with regard to their location on the positive part of the second axis. A similar pattern, yet less apparent, was also noticed for insects of the dark morphotype in comparison to the light ones.

$H_{overlap}$

Table S2. Similarities between the hypervolumes of the five functional traits depending on the five altitude levels, expressed in terms of overlapping hypervolumes ($H_{overlap}$) between the five altitude levels and computed thanks to the Sørensen index formula: **A**-over pooled data (i.e. morphotype + sex); **B**- for L-morphotype; **C**- for D-morphotype; **D**- for females and **E**- for males.

A: Pooled data

$H_{overlap}$	**11m**	**130m**	**300m**	**600m**
130m	0.419			
300m	0.017	0.144		
600m	0.002	0.047	0.545	
900m	0.160	0.301	0.509	0.380

B: L-morphotype

$H_{overlap}$	**11m**	**130m**	**300m**	**600m**
130m	0.220			
300m	0.013	0.107		
600m	0.004	0.021	0.288	
900m	0.212	0.251	0.348	0.182

C: D-morphotype

$H_{overlap}$	**11m**	**130m**	**300m**	**600m**
130m	0.388			
300m	0.004	0.063		
600m	0.000	0.019	0.263	
900m	0.023	0.116	0.398	0.377

D: F sex

$H_{overlap}$	**11m**	**130m**	**300m**	**600m**
130m	0.357			
300m	0.041	0.173		
600m	0.008	0.065	0.499	
900m	0.070	0.117	0.241	0.125

E: M sex

$H_{overlap}$	**11m**	**130m**	**300m**	**600m**
130m	0.391			
300m	0.003	0.070		
600m	0.001	0.018	0.300	
900m	0.155	0.241	0.260	0.395
